# Quantifiable ankle pump exercise monitoring for early postoperative rehabilitation after hip and knee arthroplasty: an exploratory randomized controlled trial

**DOI:** 10.3389/fresc.2026.1933740

**Published:** 2026-09-21

**Authors:** Jiquan Shen, Xinggao Wang, Changjian Zhou, Xiaoying Zhu, Huiyun Shen, Yuxuan Chen, Run Liu, Zhijun Ye, Bo Wang

**Affiliations:** 1 Department of Orthopaedics, The First Affiliated Hospital of Lishui University, Lishui Hospital of Wenzhou Medical University, Lishui People’s Hospital, Lishui, Zhejiang, China; 2 Department of Orthopedic Surgery, The Second Affiliated Hospital, Zhejiang University School of Medicine, Hangzhou, China; 3 Department of Orthopaedics, The People’s Hospital of Yunhe, Lishui, Zhejiang, China

**Keywords:** ankle pump exercise, arthroplasty, deep vein thrombosis, randomized controlled trial, rehabilitation, venous hemodynamics

## Abstract

**Background:**

Deep vein thrombosis (DVT) remains a clinically important complication after hip and knee arthroplasty. Although ankle pump exercise (APE) is widely recommended to promote venous return, adherence is often suboptimal. Quantifiable monitoring may improve exercise delivery, but its clinical value remains uncertain.

**Objective:**

To evaluate quantifiable APE monitoring for postoperative exercise adherence, DVT incidence, venous hemodynamics, and rehabilitation-related outcomes after hip or knee arthroplasty.

**Methods:**

This prospective, single-center, open-label exploratory randomized controlled trial enrolled 100 patients undergoing hip or knee arthroplasty between July and November 2023. Patients were randomized 1:1 to an experimental group receiving wearable sensor-based APE monitoring with a mobile application and real-time data transmission or a control group receiving conventional APE education and routine reminders. The registered co-primary outcomes were exercise adherence on postoperative day 7 and DVT incidence by postoperative day 14. Secondary outcomes included venous hemodynamic parameters and patient-reported outcomes, including self-efficacy assessed using the General Self-Efficacy Scale (GSES), perceived exertion assessed using the Rating of Perceived Exertion (RPE), pain assessed using the Visual Analog Scale (VAS), and patient satisfaction.

**Results:**

Exercise adherence (86% vs. 64%; absolute difference, 22.0 percentage points; 95% CI, 5.6 to 38.4; *P* = 0.011) and patient satisfaction (94% vs. 78%; absolute difference, 16.0 percentage points; 95% CI, 2.8 to 29.2; *P* = 0.021) were higher in the experimental group than in the control group. The experimental group had higher GSES scores and lower RPE and VAS scores on postoperative day 7 (all *P* < 0.05). Post-exercise femoral and popliteal venous flow velocities were also higher in the experimental group (both *P* < 0.05). DVT incidence did not differ significantly between groups (2% vs. 6%; absolute risk difference, −4.0 percentage points; 95% CI, −11.6 to 3.6; *P* = 0.617).

**Conclusions:**

Quantifiable APE monitoring was associated with improved exercise adherence and favorable short-term rehabilitation-related and venous hemodynamic outcomes. However, the study was not powered to determine whether the intervention prevents DVT.

**Clinical Trial Registration:**

https://www.chictr.org.cn/showproj.html?proj=200872, identifier, ChiCTR2300073531. Registered on July 13, 2023.

## Introduction

Hip and knee arthroplasty are effective surgical procedures for relieving pain, restoring function, and improving quality of life in patients with end-stage joint disease (1, 2). With increasing life expectancy and the growing prevalence of degenerative joint disorders, the number of arthroplasty procedures continues to rise worldwide (3, 4). Despite substantial advances in perioperative management, deep vein thrombosis (DVT) remains a clinically important postoperative complication and may lead to prolonged hospitalization, additional treatment burden, and potentially life-threatening pulmonary embolism (5, 6). Patients undergoing lower-limb arthroplasty are particularly vulnerable because of venous stasis related to surgical trauma, postoperative immobility, and activation of coagulation pathways (7, 8).

Ankle pump exercise (APE) involves flexion and extension of the ankle joint to activate the calf muscle pump and enhance venous return (9, 10). It is widely recommended as a simple, low-cost postoperative exercise to reduce venous stasis and support lower-limb circulation (11). Previous studies have suggested that APE may help reduce limb edema and improve venous hemodynamics, although its direct effect on postoperative DVT prevention remains uncertain (12, 13). Its clinical benefit depends largely on whether patients perform the exercise correctly and consistently.

In clinical practice, the effectiveness and adherence to APE can vary significantly due to patient unfamiliarity and discomfort, which may limit its preventive outcomes (14). Additionally, the time available for medical staff to provide verbal education and bedside instruction is often limited, making it challenging to ensure adequate monitoring and guidance (15). A survey of 2,203 patients by Liu et al. highlighted a notable gap between awareness and practice: despite high basic understanding (92.1%), complete exercise adherence was achieved by only 50.1%, and supervision was accepted by only 59.7% (16). Factors such as inadequate self-control, physical limitations, and cognitive impairments pose significant barriers to full implementation (17). To address these adherence challenges, recent studies have explored various quantifiable APE aids. For instance, devices utilizing localized pressure sensors or mechanical counter systems have been developed to monitor movement angles, forces, and frequencies (15, 18, 19). Although these devices can standardize movement and reduce immediate nursing workload, most operate as standalone tools without real-time integration into clinical supervision workflows.

In this study, a commercially available quantifiable APE monitoring system was integrated into routine postoperative rehabilitation. The system combined wearable motion sensing, app-based reminders, real-time data transmission, and staff feedback, thereby incorporating objective exercise monitoring into the existing clinical supervision workflow. Accordingly, the contribution of the present study was not device development or technical validation, but the clinical evaluation of a multicomponent monitoring-and-feedback strategy in patients undergoing hip or knee arthroplasty.

Therefore, this study aimed to investigate whether this quantifiable monitoring-and-feedback strategy could improve adherence to prescribed postoperative exercise in patients undergoing hip and knee arthroplasty. We further evaluated its effects on venous hemodynamics and rehabilitation-related outcomes, including self-efficacy, perceived exertion, pain, and satisfaction. DVT incidence was also assessed as a registered co-primary clinical outcome, although the study was not powered to establish a definitive effect on DVT prevention.

## Materials and methods

### Study design and participants

This was a prospective, single-center, open-label, exploratory randomized controlled trial (RCT) evaluating ankle pump exercise after hip and knee arthroplasty. The study was approved by the Lishui People's Hospital Research Ethics Committee (Approval No. 023−01) and prospectively registered in the Chinese Clinical Trial Registry on July 13, 2023 (ChiCTR2300073531). The trial was conducted in accordance with the Declaration of Helsinki and reported in line with the CONSORT statement.

No formal *a priori* sample-size or power calculation was performed. The sample size of 100 participants was determined by clinical feasibility and anticipated recruitment during the predefined study period. Therefore, this study was considered exploratory and was not powered to detect differences in relatively uncommon outcomes such as DVT. Hip and knee arthroplasty were included in the same trial because both are major lower-extremity procedures in which APE is routinely incorporated into postoperative rehabilitation. However, these populations may differ in postoperative pain, mobility, rehabilitation trajectory, and thromboembolic risk. Procedure type was therefore used as a stratification factor during randomization, and procedure-specific findings were interpreted descriptively. Because of the limited sample size within each procedure stratum, no formal interaction analysis was performed.

Patients undergoing hip or knee arthroplasty in our department between July 15 and November 30, 2023, were consecutively screened for eligibility (Figure 1). Inclusion criteria were: (1) undergoing hip or knee arthroplasty; (2) Caprini score > 3; (3) able to understand the study procedures and provide written informed consent; (4) able to complete study questionnaires independently or with minimal assistance; (5) conscious and able to communicate normally; and (6) willing and able to complete postoperative follow-up. Exclusion criteria were: (1) preoperative lower-limb DVT confirmed by color Doppler ultrasonography; (2) psychiatric disorders or cognitive impairment precluding study participation; and (3) severe visual or auditory impairment interfering with study procedures. Demographic and clinical data collected included age, sex, body mass index (BMI), procedure type, educational level, and preoperative and postoperative Caprini scores.

**Figure 1:**
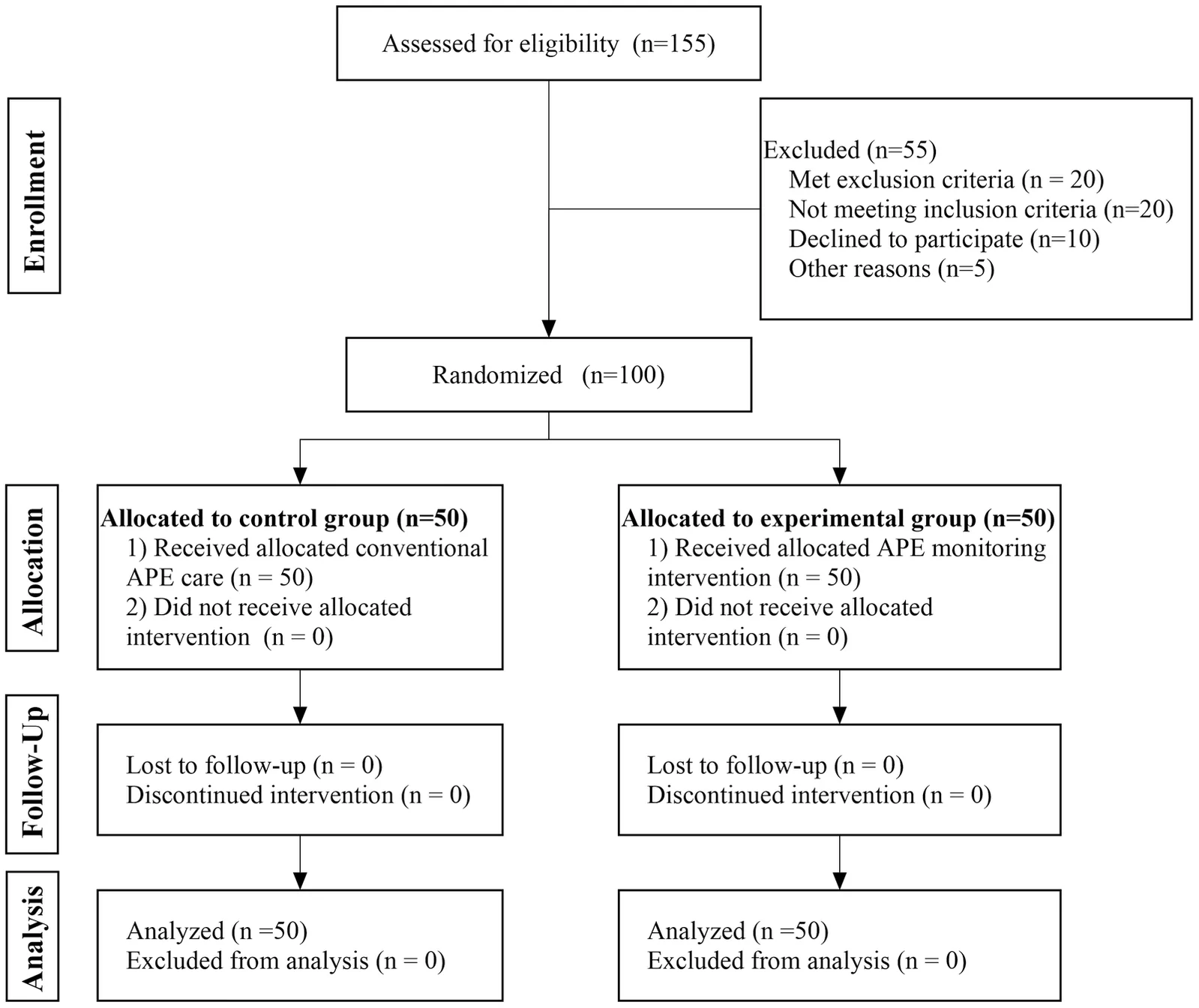
CONSORT flow diagram of participant screening, exclusion, randomization, allocation, follow-up, and analysis.

### Randomization and blinding

Eligible participants were randomized in a 1:1 ratio using stratified randomization according to procedure type (hip vs. knee arthroplasty). Within each procedure stratum, the allocation sequence was generated using computer-generated random numbers in SPSS version 22.0 (SPSS Inc., Chicago, IL, USA). Allocation concealment was maintained using sequentially numbered, opaque, sealed envelopes (SNOSE).

Because of the visible nature of the monitoring device and feedback process, blinding of participants, treating nurses, and adherence assessors was not feasible. This may have introduced performance and reporting bias, particularly for subjective outcomes. To reduce bias for objective endpoints, all Doppler ultrasound examinations were performed by sonographers blinded to group allocation, and statistical analyses were conducted by an independent blinded statistician. Data were collected by a research fellow and a professional nurse rather than the operating surgeon. Because the experimental group received app-based monitoring and supplementary feedback beyond routine supervision, the two groups were not fully attention-matched, and some degree of attention bias was unavoidable.

## Intervention

### APE training

Before the intervention, all participants received standardized APE training from healthcare professionals. Patients were instructed to perform five 3-minute sessions daily at 20-30 repetitions/min. Each repetition consisted of ankle dorsiflexion and plantarflexion to the maximum comfortable range, with each position held for 1 s. The exercise procedures under the two study conditions and representative clinical use of the monitoring device are shown in Figure 2.

**Figure 2:**
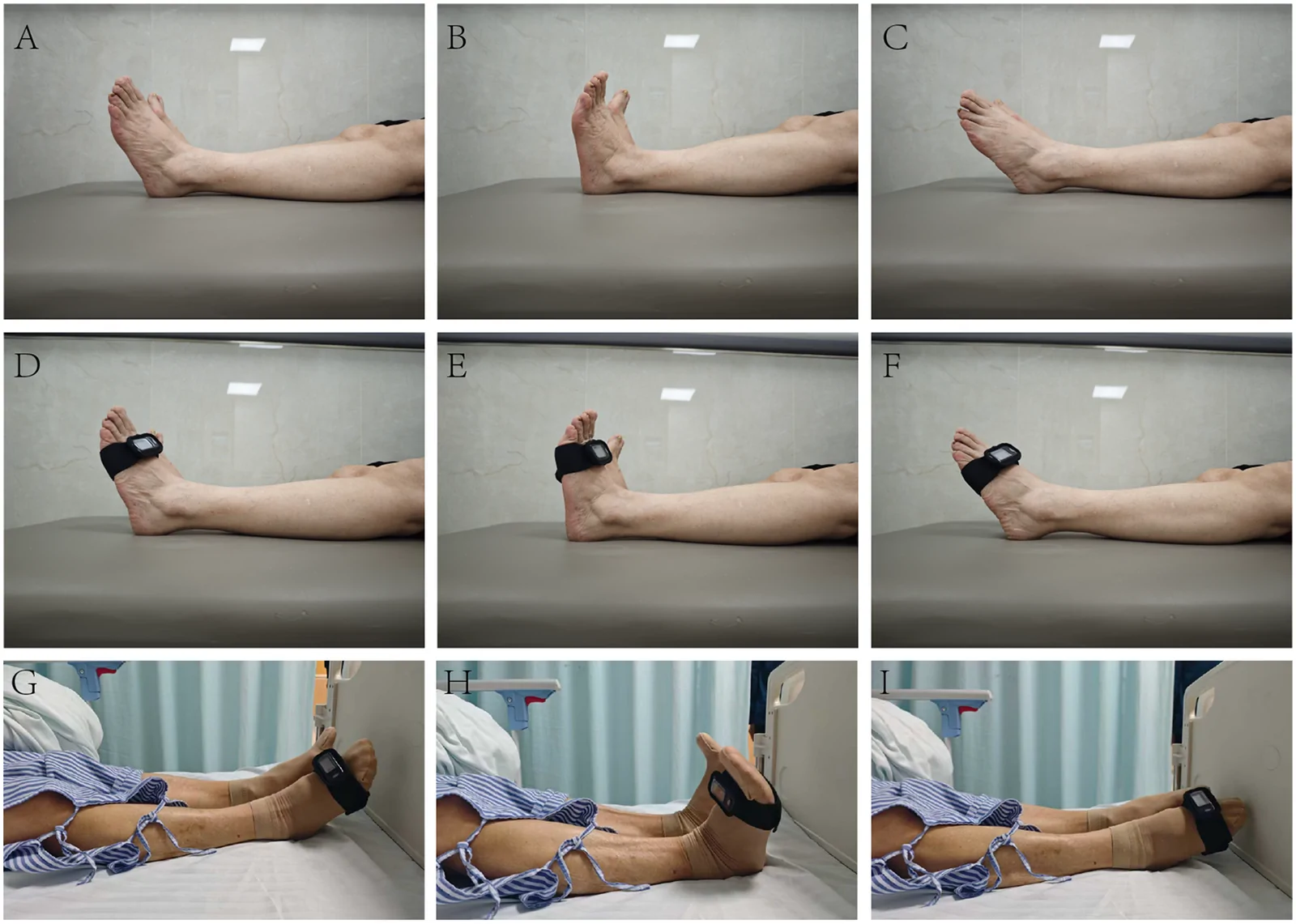
Demonstration of ankle pump exercise under the study conditions and representative clinical use of the monitoring device. **(A–C)** Standard ankle pump exercise without the monitoring device: rest position, dorsiflexion and plantarflexion, respectively; **(D–F)** ankle pump exercise performed with the monitoring device: rest position, dorsiflexion, and plantarflexion, respectively; **(G–I)** representative photographs of an actual study participant wearing the monitoring device during ankle pump exercise in the clinical setting: rest position, dorsiflexion, and plantarflexion, respectively. Panels A-F are illustrative comparisons of the two study conditions and do not indicate crossover between treatment groups; panels G-I show actual device use in a study participant.

### Control group

Patients in the control group performed standard APE. In addition to the standardized pre-enrollment instruction, they received routine supervision three times daily, corresponding to morning, midday, and evening nursing reminders delivered according to usual ward practice. Adherence was reinforced verbally during routine ward rounds, but no device-based monitoring, motion quantification, app-based prompting, or real-time exercise feedback was provided.

### Experimental group

Patients in the experimental group used the APE monitoring system (Model: ZXA-HB02, Shaoxing Zhongxinan Medical Technology Co., Ltd., China), which comprised a wearable ankle sensor connected via Bluetooth to a WeChat Mini Program. The monitoring system and representative interface views are shown in Figure 3. The wearable unit incorporated a built-in 3D acceleration sensor with triaxial gyroscopic sensing. A complete ankle-pump movement with a detected amplitude of 20°-60° was counted as one effective repetition, whereas movements outside this range were not included in the effective repetition count. The sensor algorithm and manufacturer-defined threshold were not independently validated in the present study. Exercise data were uploaded in real time through the WeChat Mini Program, allowing patients to review their exercise records and healthcare staff to monitor exercise timing and completion. The Mini Program provided three scheduled daily reminders and included supportive functions such as a countdown timer and background music. When prescribed exercise targets were not met, healthcare staff provided additional reminders or corrective guidance through WeChat. No separate measure of device-use fidelity was collected beyond the uploaded exercise records, and individual corrective contacts were not separately quantified. Thus, the intervention was a multicomponent monitoring-and-feedback strategy rather than a device-only intervention.

**Figure 3:**
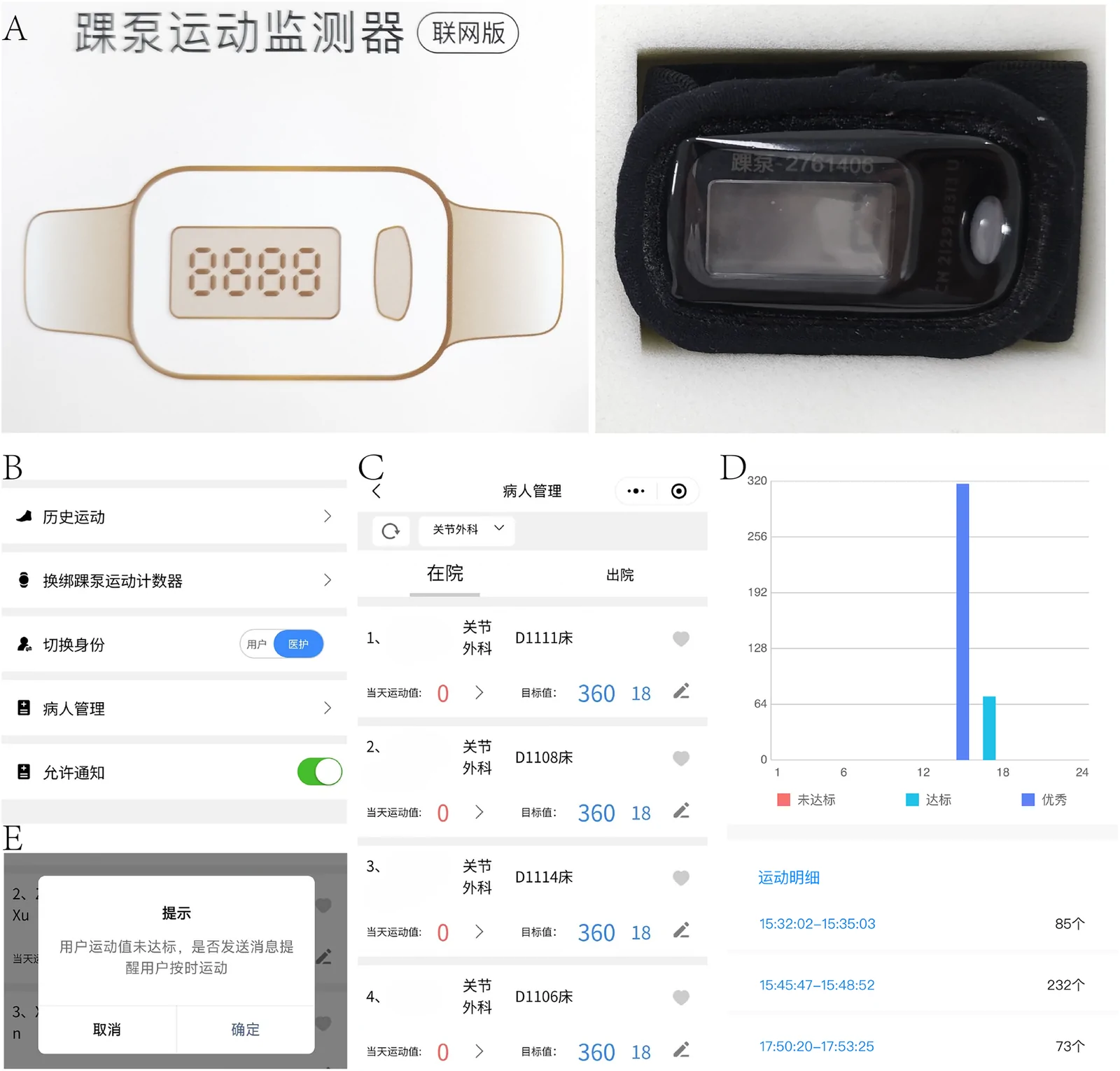
Quantifiable ankle pump exercise monitoring system and representative interface views. **(A)** Wearable ankle sensor; **(B)** Healthcare staff interface of the WeChat Mini program; **(C)** Patient-management interface showing anonymized patient information, ward/bed assignment, prescribed target values, exercise duration, and daily exercise completion; **(D)** Individual patient exercise-detail interface showing the number and timing of recorded exercise sessions; **(E)** Messaging interface used by healthcare staff to send reminders to patients who did not meet the prescribed exercise targets.

### Postoperative management

Postoperative venous thromboembolism (VTE) prophylaxis was standardized across groups. All patients received oral rivaroxaban 10 mg once daily (Bayer AG, Leverkusen, Germany, NMPA Approval No. HJ20181085) starting 12 h postoperatively; no participant required low-molecular-weight heparin as an alternative prophylactic agent. Both groups followed the same institutional postoperative pathway for pain management, early mobilization, physiotherapy, and assistive-device use, with individual adjustments made when clinically required. Patients were assisted with bedside sitting and, when appropriate, ambulation with an assistive device from postoperative day 1.

## Outcomes and assessments

### Primary outcomes

According to the prospective trial registration, exercise adherence on postoperative day 7 and DVT incidence by postoperative day 14 were prespecified co-primary outcomes. Because the study was not powered to detect differences in DVT, inference for this outcome was considered exploratory.

### Exercise adherence

Exercise adherence was assessed on postoperative day 7 using a study-specific checklist covering theoretical understanding, exercise duration, daily frequency, and exercise intensity. The checklist had not undergone formal validation. The same scoring framework was applied in both groups; however, adherence verification in the control group relied on self-report and demonstration, whereas the experimental group also incorporated device-recorded data, representing a potential source of detection bias.

Each item was scored as 2 points for meeting the standard, 1 point for nearly meeting the standard, and 0 points for not meeting the standard. Total scores were categorized as full adherence (8 points), basic adherence (5-7 points), and poor adherence (<5 points). The adherence rate was calculated with the formula: (participants with full adherence + participants with basic adherence)/total participants×100%.

### DVT incidence

Bilateral lower-extremity color Doppler ultrasonography was performed preoperatively and on postoperative day 14 by experienced vascular sonographers blinded to group allocation. The common femoral, superficial femoral, popliteal, posterior tibial, peroneal, and gastrocnemius/soleus veins were assessed bilaterally. DVT was diagnosed based on venous non-compressibility, absent flow augmentation, or direct thrombus visualization and was classified as proximal or distal and as symptomatic or screening-detected.

Additional Doppler ultrasonography was performed during the intervening postoperative period when clinically indicated, particularly in patients with marked lower-limb swelling suggestive of DVT. Routine serial screening was not performed; therefore, asymptomatic or transient interval events may have been missed. Follow-up for thromboembolic outcomes ended on postoperative day 14, and later events were not captured.

### Secondary outcomes

Secondary outcomes included venous hemodynamic parameters, patient-reported outcomes (pain, perceived exertion, and self-efficacy), and patient satisfaction.

### Venous hemodynamic outcomes

On postoperative day 7, femoral and popliteal vein diameters and blood-flow velocities on the affected side were measured at rest and immediately after 3 min of APE (Figure 4). These measurements were used to assess the short-term hemodynamic response to exercise.

**Figure 4:**
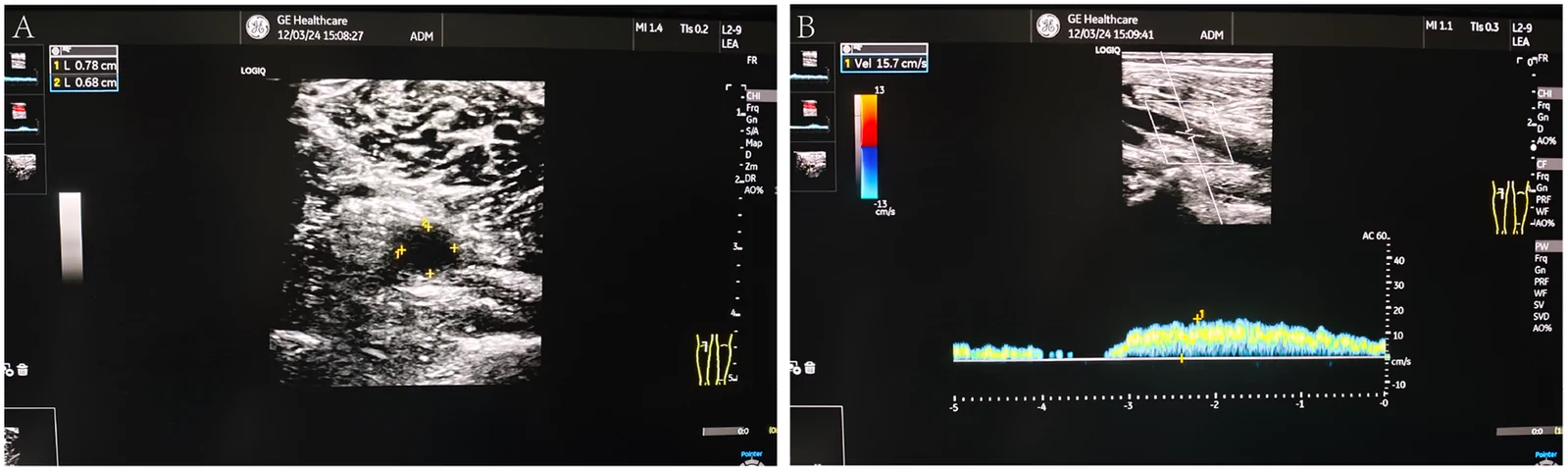
Representative Doppler ultrasonographic assessment of the popliteal vein. **(A)** Measurement of popliteal vein diameter. **(B)** Measurement of popliteal venous blood flow velocity.

### Patient-reported outcomes

Pain was assessed using the Visual Analog Scale (VAS) before the intervention and on postoperative days 1, 4, and 7, with higher scores indicating greater pain intensity. The VAS is a scale ranging from 0 to 10, where 0 indicates no pain, 1–3 represents mild pain that is tolerable and does not interfere with normal activities and sleep; 4–6 denotes moderate pain that affects sleep; and 7–10 signifies severe pain that is unbearable and disrupts sleep, appetite, and daily activities (20).

Perceived exertion was assessed on postoperative days 1, 4, and 7 using the 6–20 Rating of perceived exertion (RPE) scale (21, 22). Each point correlates with a specific level of exertion as perceived by the participant after exercising. The scale starts at 6 for “very light” exertion and extends to 20 for “very strenuous” exertion. Scores are based on the patient's immediate post-exercise sensation of fatigue in the exercising limb.

Self-efficacy was assessed using the 10-item General Self-Efficacy Scale (GSES) on the day before surgery and again on postoperative day 7 (14, 23). Scores range from 1 to 4 points, with 1 indicating “not at all true” and 4 indicating “exactly true,” reflecting the participant's confidence in their capabilities. A higher cumulative score denotes stronger self-efficacy.

### Patient satisfaction

Before discharge, patients completed an anonymous study-specific satisfaction questionnaire that had not undergone formal validation. The scoring criteria were as follows: 80–100 points for satisfaction, 60–79 points for moderate satisfaction, and below 60 points for dissatisfaction. The satisfaction rate was calculated using the formula: (Number of satisfied cases + Number of moderately satisfied cases)/Total number of cases×100%.

### Statistical analysis

Statistical analyses were performed using SPSS version 22.0 (SPSS Inc., Chicago, IL, USA). Normality was assessed using the Shapiro–Wilk test. Between-group comparisons of continuous variables were performed using independent-samples t tests or Mann–Whitney U tests, as appropriate. Categorical variables were compared using chi-square tests or Fisher's exact test when expected cell counts were <5. Absolute risk differences and relative risks with 95% confidence intervals (CIs) were reported for binary outcomes. Venous hemodynamic parameters were analyzed using repeated-measures ANOVA, with group as the between-subject factor and condition (rest vs. post-exercise) as the within-subject factor. Group, condition, and group-by-condition effects were reported with partial *η*^2^. Because the within-subject factor had only two levels, sphericity was inherently satisfied. Within-group comparisons of paired measurements were performed using paired-samples t tests. VAS, RPE, and GSES were compared between groups at the prespecified assessment points, with unadjusted mean differences and 95% CIs reported. No baseline adjustment or global multiplicity correction was applied. Accordingly, secondary outcomes and repeated time-point analyses were considered exploratory and hypothesis-generating. A two-sided *P* < 0.05 was considered statistically significant.

## Results

### Participant characteristics

Of 155 patients screened, 100 were randomized equally to the experimental and control groups. All participants received the allocated intervention and completed follow-up through postoperative day 14, with no losses to follow-up or exclusions from analysis (Figure 1). No statistically significant between-group differences were observed in the demographic and clinical characteristics shown in Table 1.

**Table 1:** Demographic and perioperative clinical characteristics by group.

Parameters	Control group (*n* = 50)	Experimental group (*n* = 50)	*P* Value
Sex (M/F)	26/24	22/28	0.423
Age (years)	73.42 ± 7.67	74.94 ± 7.51	0.319
BMI (kg/m^2^)	21.77 ± 2.75	21.54 ± 2.44	0.661
Procedure type (*n*%)			
Hip arthroplasty	30 (60%)	33 (66%)	0.534
Knee arthroplasty	20 (40%)	17 (34%)	
Educational level (*n*%)			
Junior high school and above	24 (48%)	21 (42%)	0.546
Below junior high school	26 (52%)	29 (58%)	
Caprini (score)			
Preoperative	4.86 ± 2.55	5.18 ± 2.50	0.528
Postoperative	7.60 ± 0.57	7.56 ± 0.61	0.736

Data are presented as mea*n* ± SD or n (%), as appropriate. BMI, body mass index; M, male; F, female.

### Adherence and satisfaction

As shown in Table 2, on postoperative day 7, adherence was higher in the experimental group than in the control group (86.0% vs. 64.0%; absolute difference, 22.0 percentage points; 95% CI, 5.6 to 38.4; *P* = 0.011). The satisfaction rate was also higher (94% vs. 78%; absolute difference, 16.0 percentage points; 95% CI, 2.8 to 29.2; *P* = 0.021). In descriptive subgroup analyses, adherence rates were numerically higher in the experimental group in both the hip arthroplasty (87.9% vs. 66.7%) and knee arthroplasty (82.4% vs. 60.0%) strata. Because of the limited sample size within each procedure stratum, no formal interaction analysis was prespecified or performed.

**Table 2:** Exercise adherence rate, patient satisfaction rate, and DVT incidence by group.

Parameters	Control group (*n* = 50)	Experimental group (*n* = 50)	Absolute difference, percentage points (95% CI)	*P* Value
Adherence rate (%)	64.0% (32/50)	86.0% (43/50)	22.0 (5.6 to 38.4)	0.011*
Satisfaction rate (%)	78.0% (39/50)	94.0% (47/50)	16.0 (2.8 to 29.2)	0.021*
DVT (%)	6.0% (3/50)	2.0% (1/50)	−4.0 (−11.6 to 3.6)	0.617

Data are presented as % (n/N). DVT, deep vein thrombosis; CI, confidence interval. Adherence and satisfaction were compared using chi-square tests; DVT incidence was compared using Fisher's exact test. *P < 0.05 indicates a significant difference compared with the control group. Absolute differences are expressed as experimental group minus control group in percentage points.

### Incidence of DVT

Within 14 days postoperatively, DVT occurred in 1/50 (2.0%) patients in the experimental group and 3/50 (6.0%) in the control group (absolute risk difference −4.0%, 95% CI −11.6% to 3.6%; relative risk 0.33, 95% CI 0.04 to 3.10; Fisher's exact *P* = 0.617) (Table 2). All four events were distal, asymptomatic, and detected at the scheduled postoperative day-14 screening, after completion of the day-7 venous hemodynamic assessment. No proximal DVT, symptomatic VTE, pulmonary embolism, major bleeding, or anticoagulation-related adverse event occurred. After DVT detection, affected patients received therapeutic enoxaparin (1 mg/kg every 12 h), with transition to oral rivaroxaban when clinically appropriate; therefore, the duration of enoxaparin therapy varied according to clinical management.

### Venous hemodynamic outcomes

Venous diameters did not differ significantly between groups at rest or after APE (Table 3). APE increased femoral and popliteal venous flow velocities in both groups. Significant group-by-condition interactions were observed for femoral vein velocity [F(1,98) = 12.35, *P* = 0.001, partial *η*^2^ = 0.112] and popliteal vein velocity [F(1,98) = 8.26, *P* = 0.005, partial *η*^2^ = 0.078], indicating greater exercise-related increases in the experimental group. Post-exercise femoral velocity was 23.39 ± 4.75 versus 21.28 ± 4.51 cm/s (mean difference 2.11 cm/s, 95% CI 0.27-3.95), and popliteal velocity was 18.69 ± 4.04 versus 17.14 ± 3.58 cm/s (mean difference 1.55 cm/s, 95% CI 0.04-3.06).

**Table 3:** Venous hemodynamic parameters at rest and after ankle pump exercise.

Parameters	Time	Control group (*n* = 50)	Experimental group (*n* = 50)	Mean difference, Experimental-Control (95% CI)	Between-group *P* Value
Femoral vein diameter (cm)	Rest	0.72 ± 0.11	0.71 ± 0.10	−0.01 (−0.05 to 0.03)	0.635
	After 3 min	0.74 ± 0.12	0.75 ± 0.14	0.01 (−0.04 to 0.06)	0.706
	Within-group *P* Value	0.387	0.103		
Femoral vein velocity (cm/s)	Rest	16.67 ± 3.38	16.15 ± 3.15	−0.52 (−1.82 to 0.78)	0.425
	After 3 min	21.28 ± 4.51^#^	23.39 ± 4.75^*#^	2.11 (0.27 to 3.95)	0.027
	Within-group *P* Value	<0.001	<0.001		
Popliteal vein diameter (cm)	Rest	0.70 ± 0.11	0.69 ± 0.10	−0.01 (−0.05 to 0.03)	0.635
	After 3 min	0.72 ± 0.11	0.73 ± 0.13	0.01 (−0.04 to 0.06)	0.677
	Within-group *P* Value	0.366	0.088		
Popliteal vein velocity (cm/s)	Rest	14.43 ± 3.25	14.15 ± 3.06	−0.28 (−1.53 to 0.97)	0.656
	After 3 min	17.14 ± 3.58^#^	18.69 ± 4.04^*#^	1.55 (0.04 to 3.06)	0.046
	Within-group *P* Value	<0.001	<0.001		

Data are presented as mean ± SD. Mean differences are presented as experimental group minus control group with 95% confidence intervals (CIs) and are unadjusted estimates. Between-group *P* values compare the experimental and control groups under the same measurement condition, whereas within-group *P* values compare rest and post-exercise measurements within each group. *P < 0.05 indicates a significant between-group difference. ^#^*P* < 0.05 indicates a significant within-group difference. Repeated-measures ANOVA showed significant group-by-condition interactions for femoral vein velocity [F(1,98) = 12.35, *P* = 0.001, partial *η*^2^ = 0.112] and popliteal vein velocity [F(1,98) = 8.26, *P* = 0.005, partial *η*^2^ = 0.078].

### Patient-reported outcomes

VAS scores did not differ between groups preoperatively or on postoperative day 1. The experimental group had lower VAS scores on days 4 and 7, with mean differences of −0.32 points (95% CI −0.62 to −0.02) and −0.35 points (95% CI −0.63 to −0.07), respectively (Table 4).

**Table 4:** VAS and RPE scores by group and assessment time.

Parameters	Time	Control group (*n* = 50)	Experimental group (*n* = 50)	Mean difference, Experimental-Control (95% CI)	*P* Value
VAS (score)	pre	4.53 ± 0.91	4.66 ± 0.95	0.13 (−0.24 to 0.50)	0.491
	1 d	4.22 ± 0.84	4.31 ± 0.79	0.09 (−0.23 to 0.41)	0.589
	4 d	3.46 ± 0.78	3.14 ± 0.73*	−0.32 (−0.62 to −0.02)	0.035
	7 d	2.54 ± 0.67	2.19 ± 0.74*	−0.35 (−0.63 to −0.07)	0.017
RPE (score)	1 d	16.72 ± 1.44	16.54 ± 1.37	−0.18 (−0.74 to 0.38)	0.521
	4 d	15.11 ± 1.29	14.57 ± 1.18*	−0.54 (−1.03 to −0.05)	0.031
	7 d	14.37 ± 1.05	13.82 ± 0.93*	−0.55 (−0.94 to −0.16)	0.007

Data are presented as mean ± SD. VAS, visual analog scale; RPE, Rating of Perceived Exertion; CI, confidence interval. Mean differences are presented as experimental group minus control group with 95% CIs and are unadjusted estimates. *P < 0.05 indicates significant differences from the control group.

RPE scores were similar between groups on postoperative day 1 but were lower in the experimental group on days 4 and 7, with mean differences of −0.54 points (95% CI −1.03 to −0.05) and −0.55 points (95% CI −0.94 to −0.16), respectively (Table 4).

GSES scores did not differ preoperatively. On postoperative day 7, the experimental group had a higher GSES score than the control group (25.50 ± 3.11 vs. 24.26 ± 3.07; mean difference 1.24 points, 95% CI 0.01-2.47; *P* = 0.049; Table 5).

**Table 5:** GSES scores by group and assessment time.

Time	Control group (*n* = 50)	Experimental group (*n* = 50)	Mean difference, Experimental-Control (95% CI)	Between-group *P* Value
pre	23.41 ± 3.86	23.08 ± 3.94	−0.33 (−1.88 to 1.22)	0.678
7 d	24.26 ± 3.07	25.50 ± 3.11*^#^	1.24 (0.01 to 2.47)	0.049
Within-group *P* Value	0.226	0.001		

Data are presented as mean ± SD. GSES, General Self-Efficacy Scale; CI, confidence interval. Mean differences are presented as experimental group minus control group with 95% CIs and are unadjusted estimates. *P < 0.05 indicates a significant between-group difference. ^#^*P* < 0.05 indicates a significant within-group difference (day 7 vs. preoperative).

### Safety and adverse events

No wound infection, symptomatic venous thromboembolism, major bleeding, wound hematoma, readmission, or other serious adverse event was observed during follow-up. In the experimental group, no device-related adverse events, such as skin irritation, local tenderness, swelling, or restriction of ankle movement, were reported.

## Discussion

In this exploratory randomized controlled trial, use of a quantifiable ankle pump exercise monitoring system after hip and knee arthroplasty was associated with significantly better adherence to prescribed exercise, higher patient satisfaction, improved self-efficacy, lower perceived exertion and pain, and more favorable short-term venous hemodynamic responses compared with conventional instruction alone. However, no statistically significant reduction in DVT incidence was observed during the 14-day follow-up period. These findings suggest that quantifiable monitoring may be a useful adjunct to postoperative rehabilitation after arthroplasty, although its role in thromboprophylaxis remains uncertain.

Following orthopedic surgery, the risk of DVT remains high due to hypercoagulability and extended immobility (24–26). While APE is widely used to improve venous return and reduce venous stasis, maintaining consistent adherence in routine clinical practice remains challenging (27–29). Patients frequently perform the exercise irregularly or with incorrect technique due to discomfort, lack of motivation, and limited supervision from medical staff (30, 31).

Improving adherence to postoperative preventive measures remains clinically important (32). Compared with conventional care, the monitored intervention was associated with higher adherence and patient satisfaction. Both groups received routine nursing reminders, whereas the experimental group additionally received objective monitoring and data-driven feedback. These components may have increased patient engagement and provided more structured supervision. However, the intervention was multicomponent and the groups were not fully attention-matched; therefore, the observed benefits cannot be attributed solely to the monitoring device.

Improved adherence was accompanied by more pronounced increases in femoral and popliteal venous flow velocity after exercise. This finding is physiologically plausible, as more standardized and adequately performed ankle movements may generate a more effective calf muscle pump. However, venous flow velocity is a physiological surrogate and does not establish clinical benefit or prevention of postoperative thromboembolism. The low DVT event rate may have been influenced by standardized rivaroxaban prophylaxis, while unavoidable individual variation in postoperative mobility may have affected both venous flow and DVT risk (33). Together, these factors limit attribution of any thromboembolic benefit to the APE intervention.

In the experimental group utilizing the APE monitor, patients reported improvements in self-efficacy, as well as reductions in perceived lower limb fatigue and pain scores. These findings may partly reflect the real-time feedback provided by the monitoring system, which may have supported patient self-correction and engagement during exercise. Because real-time feedback, background music, staff guidance, and other intervention components were not evaluated separately, their independent contributions to pain, perceived exertion, adherence, and satisfaction cannot be determined. Although VAS and RPE differences were statistically significant at postoperative days 4 and 7, their between-group differences were small. The VAS differences were −0.32 and −0.35 points, respectively, which were below the commonly cited 1.0 to 2.0-point minimal clinically important difference (MCID) range for pain. The corresponding RPE differences were −0.54 and −0.55 points, and no well-established MCID for postoperative RPE in this setting was available. Therefore, these findings should be interpreted as statistically detectable but clinically modest differences. Given the open-label design of the study, these findings should also be interpreted in the context of potential patient-engagement effects.

Recent studies have explored various APE aids, including pressure sensors (15), mechanical counters (14), and other quantifiable devices (18, 19). Our findings extend this literature by shifting the focus toward behavioral modification in an elective arthroplasty cohort. Consistent with previous reports (14, 18), we observed that despite significant improvements in exercise adherence and local hemodynamics, the absolute reduction in DVT incidence did not reach statistical significance. This highlights that while current technologies effectively optimize patient behavior, their definitive clinical efficacy in reducing macroscopic thromboembolic events remains exploratory. Compared with simple mechanical counters, the evaluated strategy incorporated real-time data synchronization and auditory feedback. More importantly, the present study examined its integration into clinical supervision in an elective arthroplasty cohort.

This study has several limitations. First, the single-center design and small sample size may limit generalizability, and the study was underpowered to determine an effect on DVT prevention. DVT surveillance was limited to postoperative day 14, and later thromboembolic events may therefore have been missed. Second, the open-label, multicomponent intervention included additional reminders and feedback, introducing potential performance and attention bias and preventing attribution of the observed benefits to the wearable device alone. Third, adherence was assessed differently between groups, and both adherence and satisfaction were measured using study-specific, nonvalidated instruments. Finally, postoperative continuous outcomes were not adjusted for baseline values, VAS and RPE were analyzed using scheduled time-point comparisons rather than a longitudinal mixed-effects model, and no global multiplicity adjustment was applied. Accordingly, the secondary and patient-reported findings should be considered exploratory and hypothesis-generating.

Future research should include larger multicenter trials with longer follow-up, standardized adherence measurement across groups, and prespecified clinical endpoints powered for thromboembolic outcomes. It would also be valuable to investigate whether similar monitoring systems can support broader postoperative rehabilitation programs after arthroplasty and whether cost-effectiveness justifies routine implementation.

## Conclusion

In this exploratory trial, the quantifiable APE monitoring strategy was associated with improved exercise adherence and favorable short-term patient-reported and venous hemodynamic outcomes after hip and knee arthroplasty. However, venous flow velocity is a surrogate outcome, and the study does not provide reliable evidence that the intervention prevents DVT. Larger multicenter trials with longer follow-up and adequately powered clinical endpoints are needed to determine its clinical effectiveness.

## Data Availability

The original contributions presented in the study are included in the article/Supplementary Material, further inquiries can be directed to the corresponding author.

## References

[1] Pentland V Thompson Z Dayimu A Demiris N Bohm E Campbell D. Survivorship of modern total hip replacement to 30 years: systematic review, meta-analysis, and extrapolation of global joint registry data. Lancet. (2026) 407(10531):855–66. 10.1016/s0140-6736(25)02305-0 41763743

[2] Koh CK Garceau S Grammatopoulos G Beaulé PE. Hip and knee arthroplasty in patients with obesity. J Bone Joint Surg Am. (2025) 107(21):2447–56. 10.2106/jbjs.25.00131 40938955

[3] Padhya IG Subedi S Gyawali P Regmi S. Demographics of total hip and knee arthroplasty in Nepal: an observational study. JNMA J Nepal Med Assoc. (2024) 62(279):761–4. 10.31729/jnma.8821 40656798 PMC11931332

[4] Schwartz AM Farley KX Guild GN Bradbury TL. Projections and epidemiology of revision hip and knee arthroplasty in the United States to 2030. J Arthroplasty. (2020) 35(6s):S79–85. 10.1016/j.arth.2020.02.030 32151524 PMC7239745

[5] Wolf S Barco S Di Nisio M Mahan CE Christodoulou KC Ter Haar S. Epidemiology of deep vein thrombosis. Vasa. (2024) 53(5):298–307. 10.1024/0301-1526/a001145 39206601

[6] Zhang Z Lei J Shao X Dong F Wang J Wang D. Trends in hospitalization and in-hospital mortality from VTE, 2007 to 2016, in China. Chest. (2019) 155(2):342–53. 10.1016/j.chest.2018.10.040 30419233

[7] Dubin JA Bains SS Hameed D Remily EA Moore MC Mont MA. Trends in deep vein thrombosis prophylaxis after total knee arthroplasty: 2016 to 2021. J Arthroplasty. (2024) 39(8 Suppl 1):S328–32. 10.1016/j.arth.2024.01.050 38325530

[8] Zeng Q Li Z Gui S Wu J Liu C Wang T. Prediction models for deep vein thrombosis after knee/hip arthroplasty: a systematic review and network meta-analysis. J Orthop Surg (Hong Kong). (2024) 32(2):10225536241249591. 10.1177/10225536241249591 38706263

[9] Wiercioch W Nieuwlaat R Akl EA Kunkle R Alexander KE Cuker A. Methodology for the American society of hematology VTE guidelines: current best practice, innovations, and experiences. Blood Adv. (2020) 4(10):2351–65. 10.1182/bloodadvances.2020001768 32453843 PMC7252554

[10] Liao J Wu Z. Different modes of ankle pump exercise to prevent PICC-related thrombosis through femoral vein. Phlebology. (2025) 40(1):11–4. 10.1177/02683555241269164 39079055

[11] Li H Zhang W Lu Q Wang J Zhi Y Zhang L. Which frequency of ankle pump exercise should be chosen for the prophylaxis of deep vein thrombosis? Inquiry. (2022) 59:469580221105989. 10.1177/00469580221105989 35658650 PMC9168852

[12] Liu X Hu H Jin L Lin Q Liu P Li N. Effect of postoperative ankle pump exercises on the prevention of deep vein thrombosis and venous hemodynamics following lower limb orthopedic surgery: a meta-analysis of randomized controlled trials. J Orthop Surg Res. (2025) 20(1):863. 10.1186/s13018-025-06236-7 41024203 PMC12482818

[13] He Z Liu C Ye Z Ye Z Zheng Y Zhang Z. Effects of different frequencies of ankle pump exercise on lower limb hemodynamics: a systematic review and meta-analysis. Front Physiol. (2026) 17:1880108. 10.3389/fphys.2026.1880108 42597649 PMC13470180

[14] Shi J Weng X Liu C Ge Y Chai L Ru X. The effect of the ankle pump exercise (APE) counter system assisted ankle pump motion in patients after femoral neck fracture. BMC Musculoskelet Disord. (2023) 24(1):925. 10.1186/s12891-023-06869-x 38037009 PMC10687785

[15] Xu C Zhou Y Ji J Wei C. An ankle joint flexion and extension movement-monitoring device based on pressure sensors. Micromachines (Basel). (2023) 14(12):2141. 10.3390/mi14122141 38138310 PMC10744840

[16] Liu Y Wang F Ma D Wu X Hui Z Zhang H. Views of inpatients on the prevention of venous thrombosis with ankle pump exercise: a cross-sectional survey. Phlebology. (2023) 38(1):28–35. 10.1177/02683555221142199 36433701

[17] Den Hertog R Niessen T. Taking into account patient preferences in personalised care: blending types of nursing knowledge in evidence-based practice. J Clin Nurs. (2021) 30(13-14):1904–15. 10.1111/jocn.15743 33763940

[18] Zhan Y Jiang W Wang ZC. Application of ankle pump exercise training instrument in prevention of thrombus in patients with hip fracture. J Pract Orthop. (2020) 26(08):700–702 + 706. Article in Chinese.

[19] Gao DH Li JL Pan PC Gao XX Zhou XL. Effect of quantifiable ankle pump exercise aid in preventing deep vein thrombosis of lower extremities in patients after spinal surgery. Chin Nurs Res. (2022) 36(03):522–4. Article in Chinese

[20] Faiz KW. VAS–visual analog scale. Tidsskr Nor Laegeforen. (2014) 134(3):323. 10.4045/tidsskr.13.1145 24518484

[21] Li T Yang S Hu F Geng Q Lu Q Ding J. Effects of ankle pump exercise frequency on venous hemodynamics of the lower limb. Clin Hemorheol Microcirc. (2020) 76(1):111–20. 10.3233/ch-200860 32538827

[22] Cowley N Nicholson V Timmins R Munteanu G Wood T García-Ramos A. The effects of percentage-based, rating of perceived exertion, repetitions in reserve, and velocity-based training on performance and fatigue responses. J Strength Cond Res. (2025) 39(4):e516–29. 10.1519/jsc.0000000000005026 39787033

[23] Xie Y Fu Z Li P Chen J Zhang L Liu Y. Post-traumatic growth trajectories and influencing factors in patients undergoing hip and knee arthroplasty: a prospective longitudinal study. Front Psychiatry. (2025) 16:1556829. 10.3389/fpsyt.2025.1556829 40831524 PMC12358813

[24] Lin S Alepuz A Tritsch T Schwartz G. Deep vein thrombosis prophylaxis in orthopedic surgery. Cureus. (2024) 16(2):e53726. 10.7759/cureus.53726 38455781 PMC10919879

[25] Tong K Liu H Qin J Pan Z Shangguan Y Xiao H. Current status survey of the extramural hospital management of venous thromboembolism after total hip and knee arthroplasty in China. BMC Musculoskelet Disord. (2021) 22(1):787. 10.1186/s12891-021-04663-1 34517870 PMC8438984

[26] Santana DC Emara AK Orr MN Klika AK Higuera CA Krebs VE. An update on venous thromboembolism rates and prophylaxis in hip and knee arthroplasty in 2020. Medicina (Kaunas). (2020) 56(9):416. 10.3390/medicina56090416 32824931 PMC7558636

[27] Silva KLS Figueiredo EAB Lopes CP Vianna MVA Lima VP Figueiredo PHS. The impact of exercise training on calf pump function, muscle strength, ankle range of motion, and health-related quality of life in patients with chronic venous insufficiency at different stages of severity: a systematic review. J Vasc Bras. (2021) 20:e20200125. 10.1590/1677-5449.200125 34093685 PMC8147883

[28] Wang X Tang R Zhang H Li F Wang J Li B. What frequency of ankle pump exercise is optimal to improve lower limb hemodynamics? A systematic review and network meta-analysis. Asian Nurs Res (Korean Soc Nurs Sci). (2023) 17(2):53–60. 10.1016/j.anr.2023.03.001 36933769

[29] Ge Y Xu L Ye J Qu H Fu H Ru X. Digital therapeutic intervention for venous thromboembolism prevention: efficacy evaluation of telemonitored ankle pump system in tibial plateau fracture patients. Ann Ital Chir. (2025) 96(10):1409–19. 10.62713/aic.4045 41099118

[30] Shimizu Y Kamada H Sakane M Aikawa S Mutsuzaki H Tanaka K. A novel exercise device for venous thromboembolism prophylaxis improves venous flow in bed versus ankle movement exercises in healthy volunteers. J Orthop Surg (Hong Kong). (2017) 25(3):2309499017739477. 10.1177/2309499017739477 29137566

[31] Volpe EFT Resqueti VR Da Silva AAM Gualdi LP Fregonezi GAF. Supervised exercise protocol for lower limbs in subjects with chronic venous disease: an evaluator-blinded, randomized clinical trial. Trials. (2020) 21(1):414. 10.1186/s13063-020-04314-1 32430078 PMC7238730

[32] Mefford MT Zhou H Fan D Fang MC Prasad PA Go AS. Health literacy and treatment satisfaction among patients with venous thromboembolism. J Gen Intern Med. (2023) 38(7):1585–92. 10.1007/s11606-022-07852-3 36326991 PMC10212857

[33] Ding T Chen Y Huang M Tu H Wang L. Meta-analysis of the effectiveness of ankle pump exercise combined with anticoagulant therapy for the prevention of post-operative lower extremity deep vein thrombosis. Front Neurol. (2026) 17:1899435. 10.3389/fneur.2026.1899435. 42523846 PMC13407648

